# Molecular phylogenies provide insights into the evolutionary relationships of the Spirurida (Nematoda), with special emphasis on the superfamily Physalopteroidea

**DOI:** 10.1186/s13071-025-07097-z

**Published:** 2025-11-10

**Authors:** Meng Sun, Muhammad Amjad Yousaf, Samar Harras, David I. Gibson, Hui-Xia Chen, Rasha A. Elmahy, Liang Li

**Affiliations:** 1 Hebei Collaborative Innovation Center for Eco‐Environment; College of Life Sciences, Hebei Normal University, Shijiazhuang, 050024 Hebei Province People’s Republic of China; 2 Hebei Key Laboratory of Animal Physiology, Biochemistry and Molecular Biology, Shijiazhuang, 050024 Hebei Province People’s Republic of China; 3 Zoology Department, Faculty of Science, Tanta University, Tanta, Egypt; 4 Department of Life Sciences, Natural History Museum, Cromwell Road, London, SW7 5BD UK

**Keywords:** Nematoda, Molecular phylogeny, Mitochondrial genome, Spirurida, Classification

## Abstract

**Background:**

Nematodes of the order Spirurida are of significant veterinary, medical, and economic importance. However, current knowledge of the phylogenetic relationships within the order is far from comprehensive. Moreover, the monophyly of the Physalopteroidea/Physalopteridae, and the phylogenetic relationships of its three component subfamilies, remain uncertain due to inadequate sequence data.

**Methods:**

The nuclear small ribosomal subunit (18S rRNA) and large ribosomal subunit (28S rRNA), plus the complete mitochondrial genomes of two physalopterid species, *Thubunaea pudica* (Thubunaeinae) and *Abbreviata varani* (Physalopterinae), are presented for the first time. Phylogenetic analyses of the Spirurida were performed using maximum likelihood and Bayesian inference on the basis of different concatenated datasets involving the most comprehensive subfamily-level taxon sampling of the superfamily Physalopteroidea to date to provide an initial understanding of the evolutionary relationships of major superfamilies within the order, with special emphasis on the Physalopteroidea/Physalopteridae.

**Results:**

The complete mitogenomes of *T. pudica* and *A. varani* are 13,645 bp and 13,730 bp in length, which both contain 36 genes and belong to the GA9 type gene arrangement. Molecular phylogenies based on different datasets all support a close affinity between the superfamilies Camallanoidea and Dracunculoidea. Our phylogenetic results also showed that the representatives of the Physalopteridae did not form a monophyletic group. The representative of the subfamily Proleptinae clustered together with the tetramerid species *Crassicauda magna*, and the representative of the subfamily Thubunaeinae formed a sister relationship with species of the subfamily Physalopterinae.

**Conclusions:**

The mitogenome of *T. pudica* is the first for the subfamily Thubunaeinae, and that of *A. varani* is also the first for the genus *Abbreviata*. Molecular phylogenetic results suggest that the subfamily Proleptinae should be elevated to full family level, i.e., Proleptidae stat. nov., and that the genus *Crassicauda* (Habronematoidea: Tetrameridae) should be assigned to Proleptidae stat. nov. Our results also indicate that the Physalopteridae *sensu stricto* currently comprises two subfamilies, the Thubunaeinae and the Physalopterinae, and that the genus *Skrjabinoptera* should be transferred from the Physalopterinae to the Thubunaeinae.

**Graphical Abstract:**

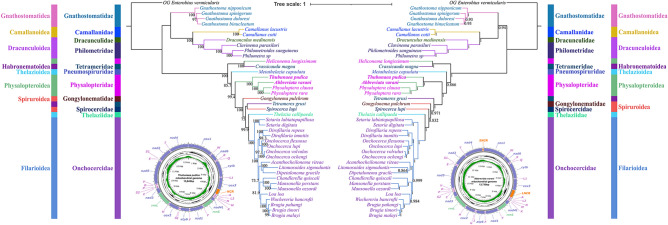

**Supplementary Information:**

The online version contains supplementary material available at 10.1186/s13071-025-07097-z.

## Background

The order Spirurida is a diverse and important group of zooparasitic nematodes, which occur in various tissues and organs of all major vertebrate groups, including humans [1–3]. Some species of the Spirurida can cause disease in domestic animals, wildlife, and humans (e.g., Dracunculiasis, Gnathostomiasis, Filariasis, Thelaziasis, and Gongylonemiasis) [3–7]. According to traditional classifications [8–10], the order Spirurida is divided into 12 superfamilies, namely Acuarioidea, Aproctoidea, Camallanoidea, Diplotriaenoidea, Dracunculoidea, Filarioidea, Habronematoidea, Gnathostomatoidea, Physalopteroidea, Rictularioidea, Spiruroidea, and Thelazioidea. However, the phylogenetic relationships of these superfamilies within the Spirurida are still poorly understood [11].

The superfamily Physalopteroidea includes only the single family Physalopteridae [10, 12], which comprises a large group of zooparasitic nematodes, including more than 300 described species occurring in all major lineages of vertebrates [10, 13, 14]. The Physalopteridae currently contains three subfamilies, namely the Physalopterinae, Proleptinae, and Thubunaeinae [10, 14]. Among these, species of the Proleptinae parasitize only teleosts and elasmobranchs [15–17], whereas members of the Thubunaeinae are obligatory parasites of reptiles [10, 18, 19]. The subfamily Physalopterinae has an extremely high diversity, including approximately 230 nominal species, occurring in the alimentary canal of amphibians, reptiles, birds, and mammals [10, 13, 14, 20–23]. However, the monophyly of the Physalopteroidea/Physalopteridae, and the phylogenetic relationships of its three component subfamilies remain uncertain [23], due to inadequate sequence data.

The subfamily Thubunaeinae is the key group for solving the evolutionary relationships of the Physalopteroidea/Physalopteridae, which currently contains only two genera, namely *Thubunaea* and *Physalopteroides* [10, 14]. However, the genetic data base for the Thubunaeinae remains very limited, since only a single, unidentified species of *Physalopteroides* has been genetically sequenced for the small ribosomal DNA (18S). No data on the mitochondrial genome of members of the Thubunaeinae have previously been reported.

In this study, the complete mitochondrial genomes of *Thubunaea pudica* Seurat, 1914 (Thubunaeinae) and *Abbreviata varani* (Parona, 1889) (Physalopterinae) were sequenced and annotated for the first time to reveal the characterization of physalopterid mitogenomes. Moreover, to further investigate the phylogenetic relationships of the order Spirurida, with special emphasis on the Physalopteroidea/Physalopteridae, phylogenetic analyses based on different concatenated datasets [nucleotide sequences of 18S (small ribosomal DNA) + *cox*1 (cytochrome c oxidase subunit I), amino acid (AA) sequences of 12 protein-coding genes (PCGs) of mitogenomes, and nucleotide sequences of 18S + 28S (large ribosomal DNA) + 12 PCGs of mitogenomes] were performed using maximum likelihood and Bayesian inference.

## Methods

### Parasite collection and species identification

The specimens of *Thubunaea pudica* used in this study were collected from the gastrointestinal tract of *Trapelus mutabilis* (Squamata: Agamidae) captured near El Dabaa (31°20′N, 28°26′E), Egypt. Nematodes of *Abbreviata varani* were collected from the gastrointestinal tract of the Bengal monitor *Varanus bengalensis* (Lacertiformes: Varanidae) at Sheringal (35°27′N, 71°99′E), Upper Dir Shahi District, Pakistan. Nematode specimens were fixed and preserved in 70% ethanol for morphological studies.

**Fig. 1:**
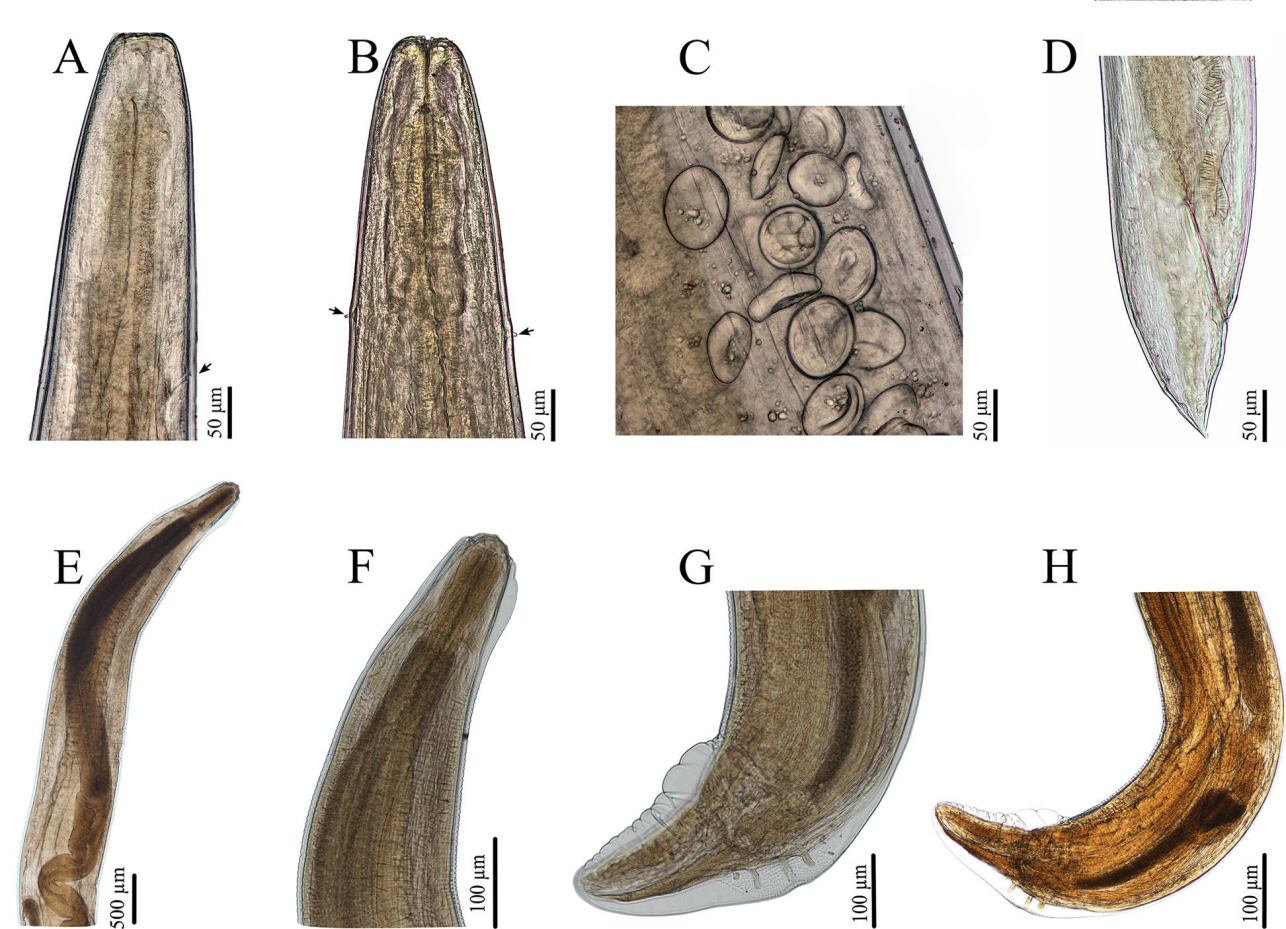
Photomicrographs of *Thubunaea pudica* (**A**–**D**) and *Abbreviata varani* (**E**–**H**). **A** anterior region of female (excretory pore arrowed), lateral view; (**B**) anterior region of female (deirids arrowed), dorsal view; (**C**) eggs; (**D**) posterior end of female, lateral view; (**E**, **F**) anterior region of male, lateral view; (**G**) posterior end of male, ventral view; (**H**) posterior end of male, lateral view

**Fig. 2:**
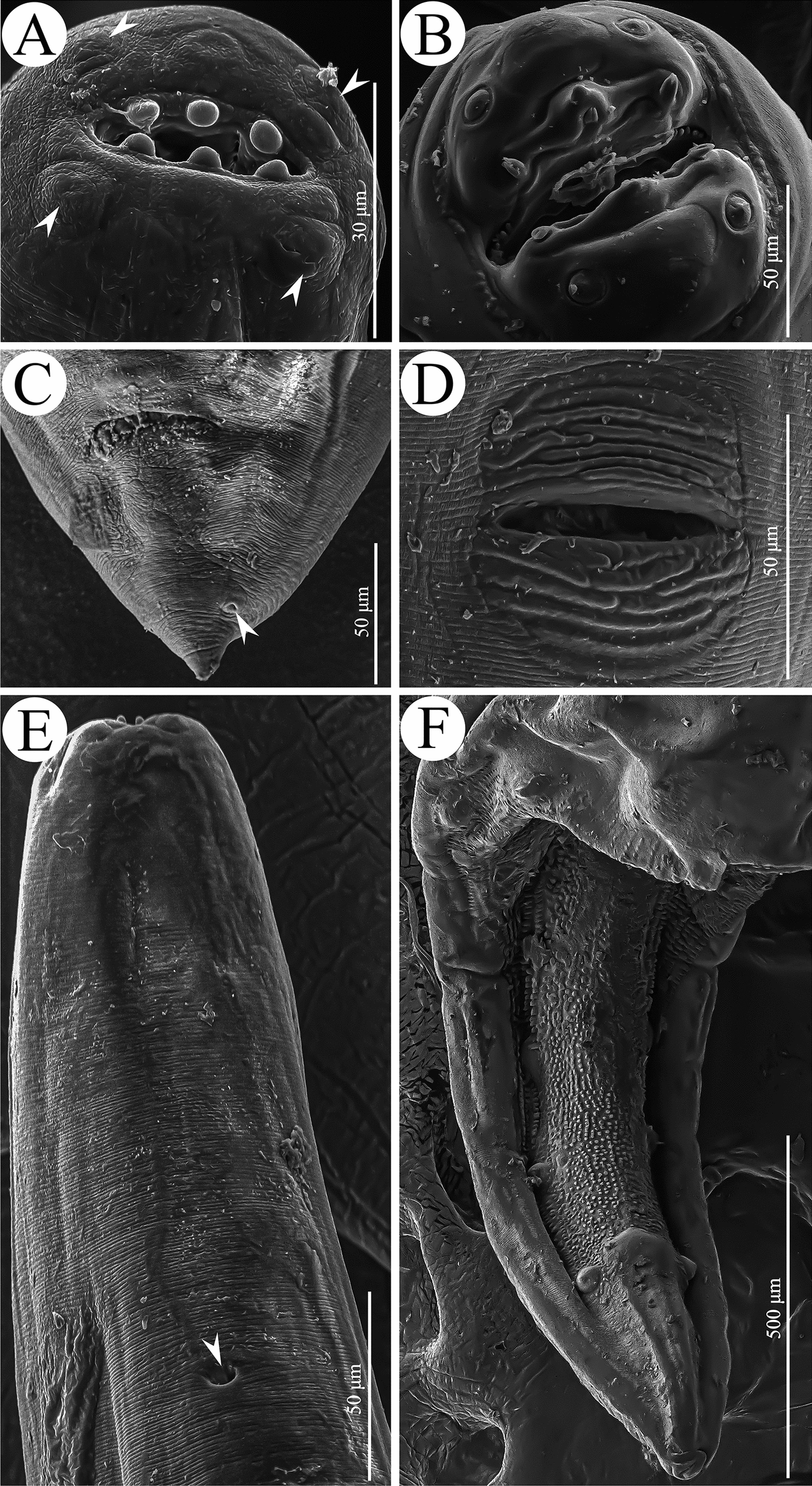
Scanning electron micrographs of *Thubunaea pudica* (**A**, **C**–**E**) and *Abbreviata varani* (**B**, **F**). **A** cephalic region of female (cephalic papillae arrowed), apical view; (**B**) cephalic region of male, apical view; (**C**) tail of female (phasmid arrowed), ventral view; (**D**) region of vulva; (**E**) anterior region of male (excretory pore arrowed), ventral view; (**F**) posterior end of male, ventral view

The morphology of these nematode specimens was observed using light and scanning electron microscopy (LM and SEM) (Figs. 1, 2), which were identified as *T. pudica* and *A. varani* according to previous studies [13, 24]. Voucher specimens of *T. pudica* (HBNU–N–R20240815YL) and *A. varani* (HBNU–N–R20250530YL) were deposited in the College of Life Sciences, Hebei Normal University, Hebei Province, China.

### Molecular procedures

Genomic DNA was extracted from two females of *T. pudica* and one female of *A. varani* using the TIANGEN Genomic DNA Extraction Kit (TIANGEN Biotech Co. Ltd., Beijing, China) following the manufacturer’s protocol. Polymerase chain reaction (PCR) was employed to amplify different target regions using the primers and cycling conditions provided in Supplementary file: Table S1. PCR products were checked on GoldView-stained 1.5% agarose gels and purified using the Column PCR Product Purification Kit [Sangon Biotech (Shanghai) Co. Ltd.]. Sequencing of each sample was carried out for both strands. The 18S, 28S, and *cox*1 sequence data of *T. pudica* and *A. varani* obtained were deposited in the GenBank database (http://www.ncbi.nlm.nih.gov) under the following accession numbers (*T. pudica*: PV818394 and PV818395 for 18S, PV916231 and PV916232 for 28S, PV819810 for *cox1*; *A. varani*: PV864872 for 18S, PV818399 for 28S, PV888636 for *cox1*).

### Mitochondrial genome sequencing, assembly, and annotation

A total of 50 GB of clean genomic data for each species were generated using the Pair-End 150 sequencing method on the Illumina NovaSeq 6000 platform (Illumina, Inc., San Diego, CA, USA) by Novogene Technology Co. Ltd. (Tianjin, China). The complete mitochondrial genomes of *T. pudica* and *A. varani* were assembled, annotated, and analyzed using different software programs or tools according to the methods and procedures provided in previous studies [25–30]. The mitogenomes of *T. pudica* and *A. varani* generated in this study were deposited in the GenBank database (https://www.ncbi.nlm.nih.gov/) under accession numbers (PV925727 for *T. pudica*, PV925728 for *A. varani*).

### Phylogenetic analyses

Phylogenetic analyses were performed using maximum likelihood (ML) with IQTREE v2.1.2 [31], and Bayesian inference (BI) under MrBayes 3.2.7 [32] on the basis of the concatenated datasets, including the nucleotide sequences of 18S + *cox*1, amino acid (AA) sequences of the 12 protein-coding genes (PCGs), and the nucleotide sequences 18S + 28S + 12 PCGs. The pinworm *Enterobius vermicularis* (Oxyurida: Oxyuridae) was chosen as the outgroup. Detailed information for representatives of the ingroup is provided in Supplementary file: Table S2.

Sequences were aligned using the MAFFT v7.313 multiple sequence alignment program under the E-INS-I iterative refinement method [33]. Poorly aligned regions were excluded using BMGE v1.12 (*h* = 0.4) [34]. Substitution models were compared and selected using the Bayesian information criterion (BIC) under ModelFinder [35]. For BI analyses, the optimal amino acid or nucleotide substitution models were identified as follows: the JTT + F + I + G4 model for the amino acid (AA) sequences of the 12 PCGs, and the WAG + F + I + G4 model for both of the nucleotide sequences of 18S + *cox*1 and 18S + 28S + 12 PCGs. The optimal amino acid or nucleotide substitution model selected for the ML analyses are provided in Supplementary file: Table S3. Reliabilities for the ML inference were tested using 1000 bootstrap replications, and the BIC analysis was run for 5 × 10^6^ MCMC generations.

## Results

### Complete mitochondrial genomes of *Thubunaea pudica* and *Abbreviata varani*

**Fig. 3:**
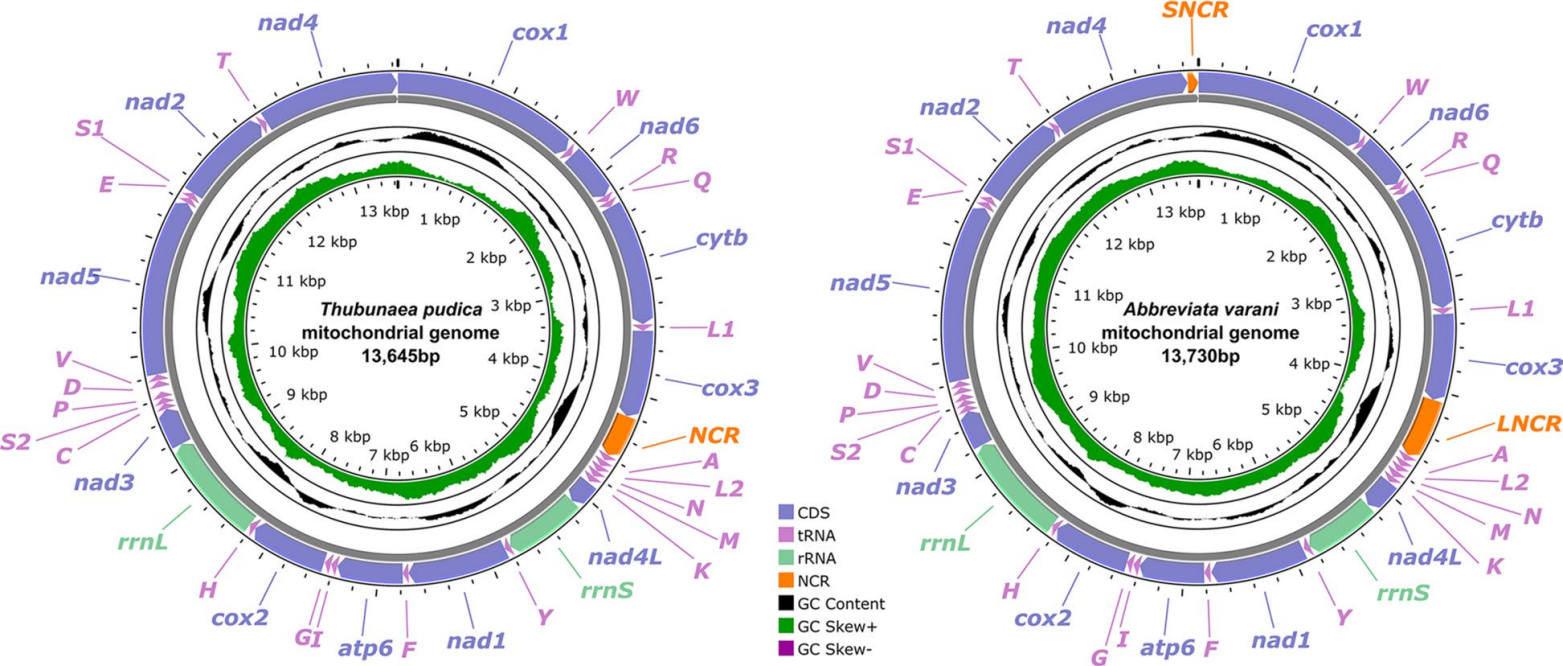
Gene map of the mitochondrial genomes of *Thubunaea pudica* and *Abbreviata varani*. All 22 tRNA genes are indicated by a single-letter code, with numerical suffixes distinguishing each of the two tRNA genes, serine and leucine. All genes are transcribed clockwise on the same strand. The outermost circle shows the GC content, while the innermost circle displays the GC skew

The length of the complete mitochondrial genome of *T. pudica* is 13,645 bp, and that of *A. varani* is 13,730 bp. Both mitogenomes contain 36 genes, including 12 protein-coding genes (PCGs) (*cox*1–3, *cytb*, *nad*1–6, *nad*4L, and *atp*6; lacking *atp*8), 22 tRNA genes, and two rRNA genes (*rrn*L and *rrn*S) (Fig. 3, Supplementary file: Table S4). All genes of the mitogenomes of *T. pudica* and *A. varani* are transcribed from the same strand in the same direction. There is only one noncoding region (NCR) in the mitogenome of *T. pudica* (383 bp, between *cox*3 and *trn*A), but two noncoding regions are present in the mitogenome of *A. varani* (LNCR is 544 bp, between *cox*3 and *trn*A; SNCR is only 96 bp, between *nad4* and *cox*1) (Fig. 3, Supplementary file: Table S4). The overall A + T content in the mitogenomes of *T. pudica* and *A. varani* are 75.5% and 74.7%, respectively, both displaying a strong A + T bias. Details of the nucleotide contents of the mitogenomes of *T. pudica* and *A. varani* are provided in Supplementary files: Tables S4, S5.

**Fig. 4:**
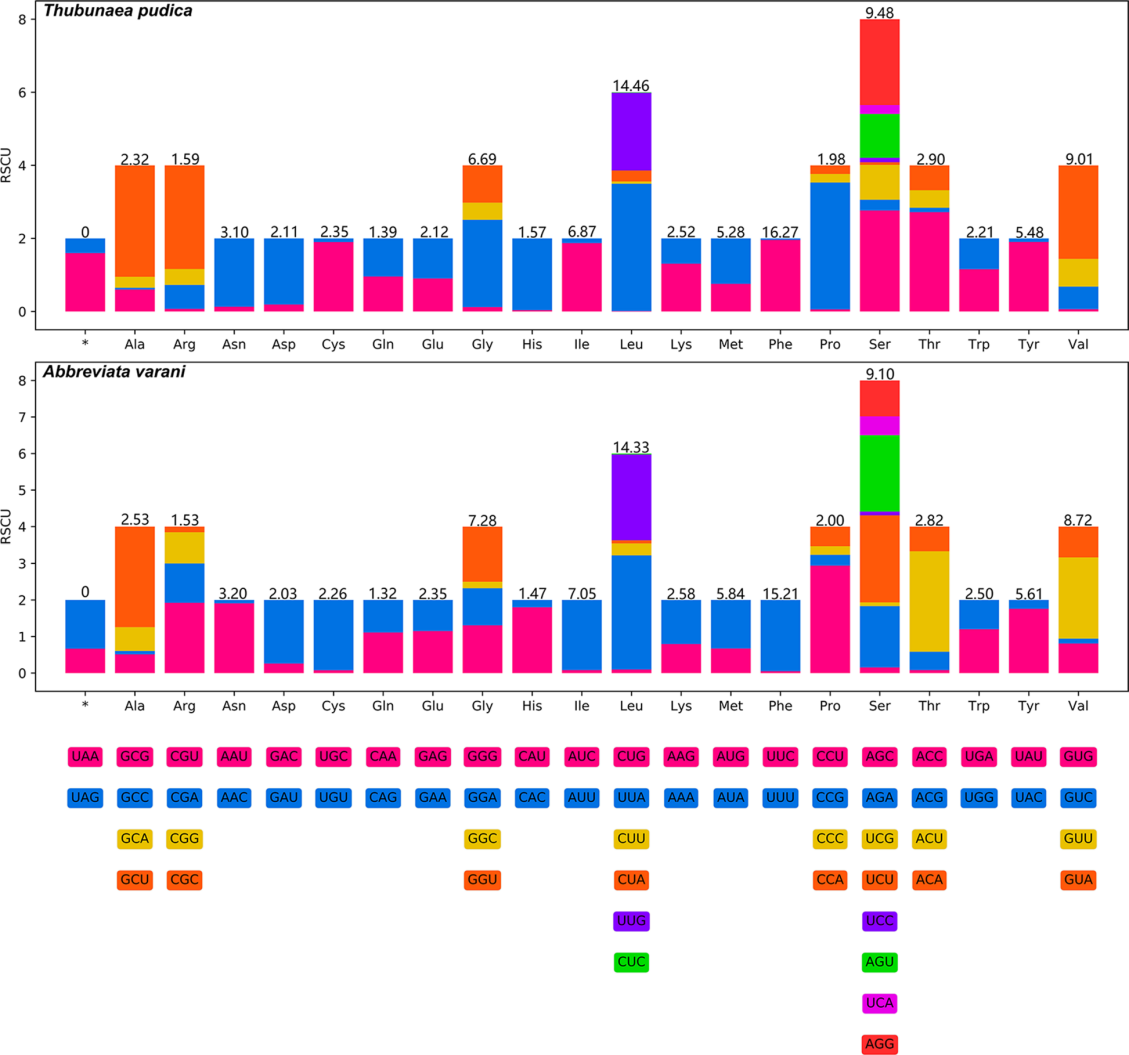
Relative synonymous codon usage (RSCU) of the mitogenomes of *Thubunaea pudica* and *Abbreviata varani*. Codon families (in alphabetical order) are labeled below the horizontal axis. Values above each bar represent amino acid usage in percentages

The 12 PCGs of the mitogenome of *T. pudica* have 10,350 bp and encode 3449 amino acids (excluding the stop codons), whereas the 12 PCGs of the mitogenome of *A. varani* have 10,219 bp and encode 3405 amino acids (excluding the stop codons) (Supplementary file: Table S4). The size of each of the 12 PCGs and their components and usages of the codons in the mitogenomes of *T. pudica* and *A. varani* are provided in Fig. 4 and Supplementary file: Table S4. A total of 22 tRNAs were identified in both mitogenomes of *T. pudica* and *A. varani*, ranging from 51 to 60 bases in size. The size for each tRNA is provided in Supplementary file: Table S4.

The 36 gene arrangement in the mitogenomes of *T. pudica* and *A. varani* are identical, both belonging to the GA9 type arrangement in the following order: *cox1*, *trnW*, *nad6*, *trnR*, *trnQ*, *cytb*, *trnL1*, *cox3, trnA, trnL2, trnN, trnM, trnK, nad4L, rrnS, trnY, nad1, trnF, atp6, trnI, trnG, cox2, trnH, rrnL, nad3, trnC, trnS2, trnP, trnD, trnV, nad5, trnE, trnS1, nad2, trnT, nad4* (Fig. 3).

### Phylogenetic relationships of taxa within the Spirurida

**Fig. 5:**
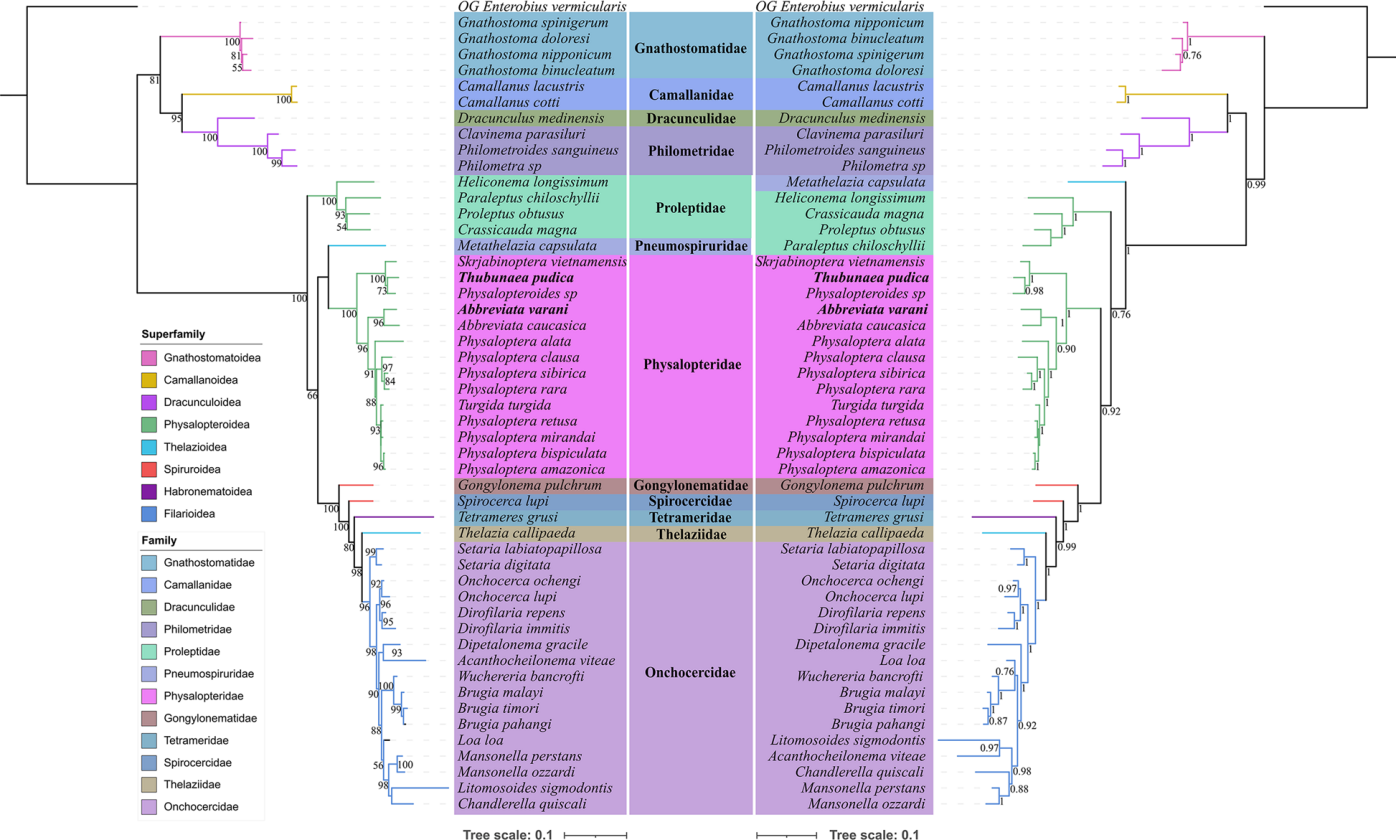
Phylogenetic analyses of the Spirurida based on the nucleotide sequences of the 18S + *cox1*, using maximum likelihood (ML) (left side) and Bayesian inference (BI) (right side) methods. *Enterobius vermicularis* (Spirurida: Oxyuridae) was used as the outgroup. Different families were showed using different background colors of species name. Different superfamilies were shown using different line colors of branches. The bootstrap support (BS) values ≥ 50 in the ML tree and Bayesian posterior probability (BPP) values ≥ 0.70 in the BI tree were shown

Phylogenetic results based on the 18S + *cox1* sequence data, using both ML and BI methods, have similar topologies (Fig. 5). In the BI tree of the18S + *cox1* sequence data, species of the superfamily Gnathostomatoidea are the most basal and formed a sister relationship with the remaining representatives of the Spirurida [versus Gnathostomatoidea + (Camallanoidea + Dracunculoidea) in the ML tree]. The representatives of Camallanoidea and Dracunculoidea clustered together in both BI and ML trees. The representatives of the Physalopteroidea/Physalopteridae did not form a monophyletic group, and the tetramerid species *Crassicauda magna* nested with species of the subfamily Proleptinae in both BI and ML trees. The other representatives of the Physalopteridae were divided into two lineages (*Thubunaea pudica*, *Skrjabinoptera vietnamensis*, and *Physalopteroides* sp. formed a lineage representing the subfamily Thubunaeinae, whereas *Turgida turgida*, *Physaloptera* spp., and *Abbreviata* spp. formed the other lineage representing the subfamily Physalopterinae). Both BI and ML trees showed that the superfamilies Thelazioidea (including species of the Pneumospiruridae and Thelaziidae) and Spiruroidea (including representatives of the Gongylonematidae and Spirocercidae) are not monophyletic. The phylogenetic position of the family Pneumospiruridae (*Metathelazia capsulata*) is different in the BI and ML trees. The family Thelaziidae displayed a sister relationship with the Onchocercidae (Filarioidea) in both BI and ML trees.

**Fig. 6:**
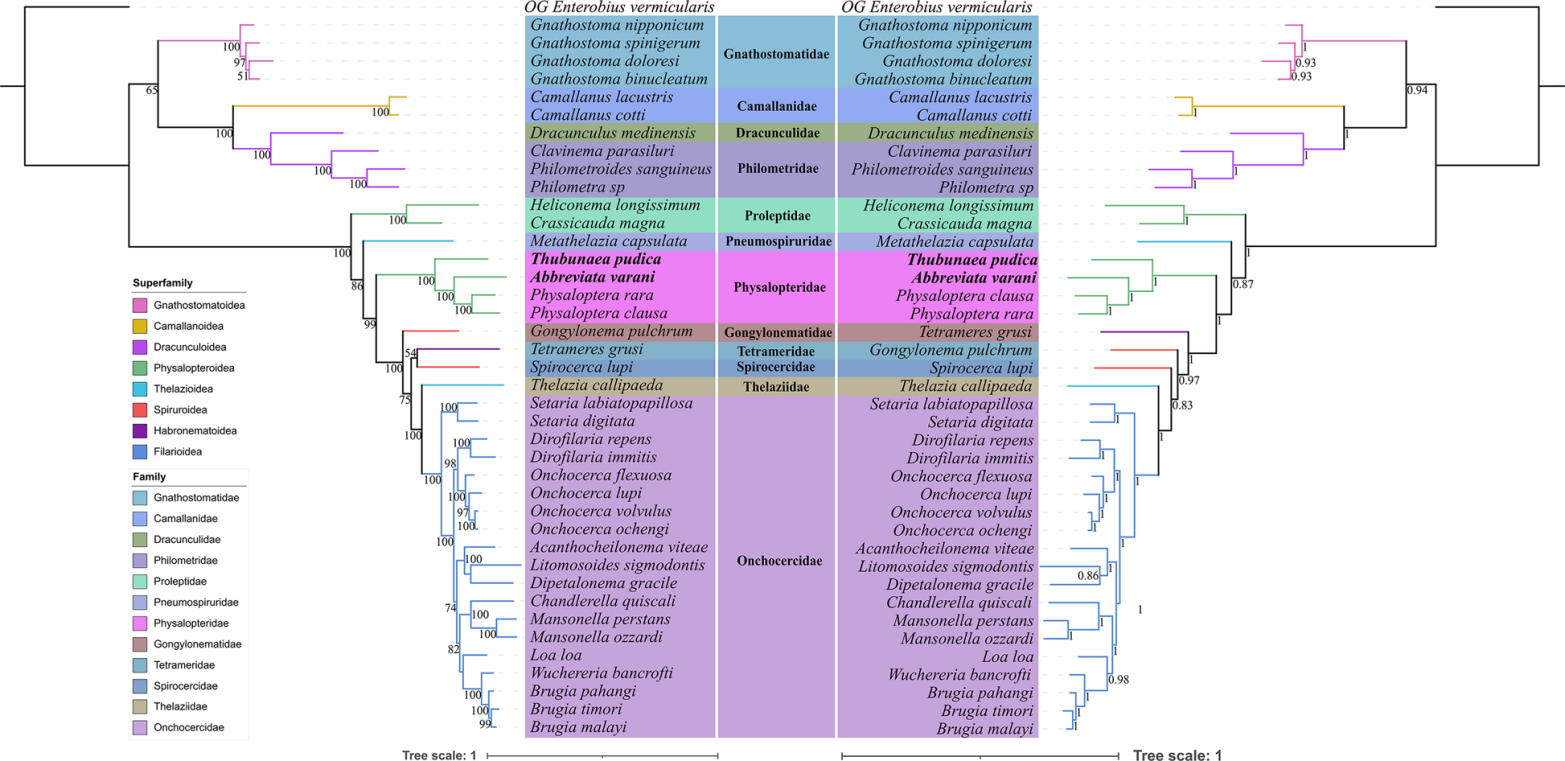
Phylogenetic analyses of the Spirurida based on the amino acid (AA) sequences of 12 protein-coding genes (PCGs) of the mitogenomes, using maximum likelihood (ML) (left side) and Bayesian inference (BI) (right side) methods. *Enterobius vermicularis* (Spirurida: Oxyuridae) was used as the outgroup. Different families were shown using different background colors of species name. Different superfamilies were shown using different line colors of branches. The bootstrap support (BS) values ≥ 50 in the ML tree and Bayesian posterior probability (BPP) values ≥ 0.70 in the BI tree were shown

**Fig. 7:**
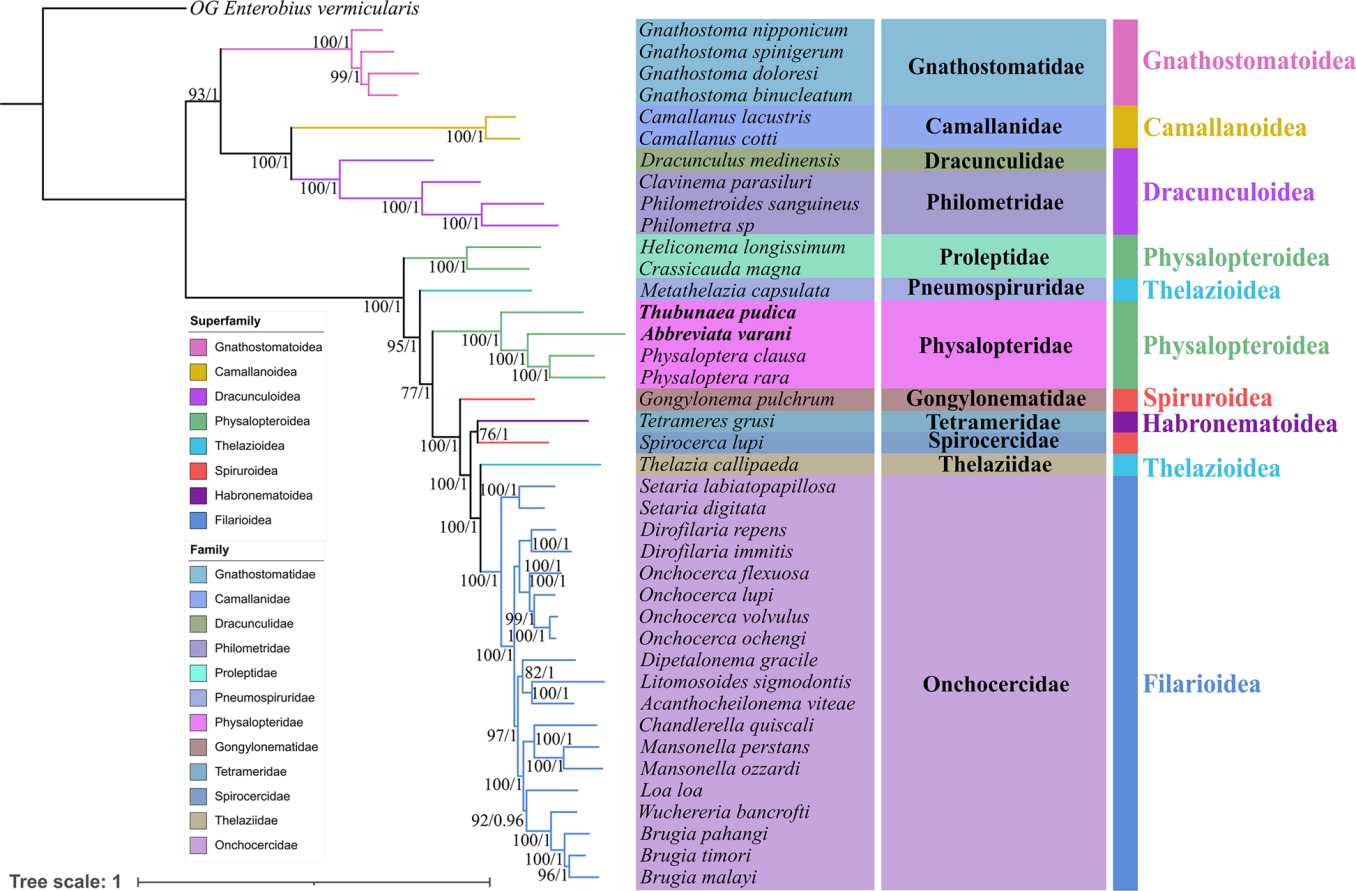
Phylogenetic of the Spirurida based on the nucleotide sequences of the 18S + 28S + 12 PCGs of the mitogenomes, using maximum likelihood (ML) and Bayesian inference (BI) methods. *Enterobius vermicularis* (Spirurida: Oxyuridae) was used as the outgroup. Different families were shown using different background colors of species name. Different superfamilies were shown using different line colors of branches. The bootstrap support (BS) values ≥ 50 in the ML tree and Bayesian posterior probability (BPP) values ≥ 0.70 in the BI tree were shown

Phylogenetic trees constructed using both ML and BI methods on the basis of the concatenated amino acid (AA) sequences of the 12 protein-coding genes (PCGs), and the concatenated nucleotide sequences of the 18S + 28S + 12 PCGs, had almost identical topologies (Figs. 6, 7). All of the phylogenetic results showed that the superfamilies Camallanoidea and Dracunculoidea have a sister relationship and clustered together with the Gnathostomatoidea; these results are identical to the ML tree of the 18S + *cox1* data. Our phylogenetic results also showed that the representatives of the Physalopteridae did not form a monophyletic group. The representative of the subfamily Proleptinae (*Heliconema longissimum*), clustered together with the tetramerid species *Crassicauda magna* and the representative of the subfamily Thubunaeinae (*Thubunaea pudica)*, formed a sister relationship with species of the subfamily Physalopterinae (*Turgida turgida*, *Physaloptera clausa*, *P. rara* and *Abbreviata varani*) in the phylogenetic trees with strong support. BI and ML results of the amino acid sequences of the 12 PCGs and the nucleotide sequences of the 18S + 28S + 12 PCGs all showed that the superfamilies Thelazioidea (Pneumospiruridae and Thelaziidae) and Spiruroidea (Gongylonematidae and Spirocercidae) are not monophyletic, outcomes that are in accord with those of the 18S + *cox1* data. The phylogenetic position of the family Pneumospiruridae (*Metathelazia capsulata*) in the topologies of the amino acid sequences of 12 PCGs and the nucleotide sequences of 18S + 28S + 12 PCGs is identical to that of the ML tree of 18S + *cox1* data. The present results also show that the family Thelaziidae (Thelazioidea) is sister to the Onchocercidae (Filarioidea) with strong support.

## Discussion

Our current knowledge of the mitochondrial genomes of the Physalopteroidea/Physalopteridae is limited. To date, only three species belonging to two genera (*Physaloptera* and *Heliconema*), representing the two subfamilies Physalopterinae and Proleptinae, have had their mitochondrial genomes sequenced [36]. This study provides the first mitogenomic data for the subfamily Thubunaeinae and the genus *Abbreviata* (Physalopterinae). Comparative mitogenomics reveal that the size and overall A + T content in the mitogenomes of *T. pudica* and *A. varani* (13,645 and 13,730 bp in size, 75.5%, and 74.7% overall A + T content) are similar to those of *Physaloptera rara*, *P. clausa* and *Heliconema longissimum* (13,610–13,735 bp in size, and 72.5–79.1% overall A + T content) [36]. In the order Spirurida, a total of 38 species are available in terms of their complete mitochondrial genomes (Supplementary file: Table S2). The commonest gene arrangement for the mitogenomes of species of this order is type GA9 [37]. The gene arrangement of the mitogenomes of *T. pudica* and *A. varani* (GA9 type) is identical to that of *Physaloptera* spp., and most of species of the Filarioidea, Thelazioidea, Spiruroidea, and Habronematoidea, but different to that of the Gnathostomatoidea (GA23 type, GA34 type), Camallanoidea (GA40 type, GA48 type), and Dracunculoidea (GA14 type, GA50 type, GA55 type, GA56 type) (Supplementary file: Table S2).

The present molecular phylogenies based on different datasets all support a close affinity between the superfamilies Camallanoidea and Dracunculoidea and are concordant with the traditional view [8–10] and some phylogenetic studies [38–42]. The superfamily Gnathostomatoidea is a particular and primitive group of the Spirurida [8]. Although some molecular phylogenetic studies have made efforts to clarify the phylogenetic position of the Gnathostomatoidea on the basis of different genetic data, the evolutionary relationships of this superfamily in relation to other groups within the Spirurida are still under discussion [40–46]. The present phylogenetic results from BI tree of 18S + *cox1* data indicate that the Gnathostomatoidea forms a sister relationship with the Camallanoidea + Dracunculoidea. This “unexpected” phylogenetic position for the Gnathostomatoidea has not previously been suggested. However, it seems to be not difficult to understand the proposed phylogenetic relationships in this study when we consider that the representatives of Gnathostomatoidea, Camallanoidea, and Dracunculoidea all utilize copepods as their intermediate host, which ingest the free larvae in water [3]. The similar life cycle of three parasite groups appears to support their close affinity.

Our phylogenetic analyses included the most comprehensive taxon sampling of the Physalopteroidea/Physalopteridae to date, and all of the results, based on different datasets, fail to support the monophyly of this group. These results differ from the previous studies [8–11, 14, 39, 41], but agree with some phylogenetic conclusions [38, 47]. Our findings suggest the elevation of the subfamily Proleptinae to full family status, as the Proleptidae stat. nov., and also indicate that the tetramerid genus *Crassicauda* (Habronematoidea) should be transferred to the Proleptidae, which are consistent with the recent mitogenomic phylogeny [48]. However, the previous phylogenetic studies based on the single 18S rRNA showed that *Crassicauda* has a close affinity with the family Acuariidae (Acuarioidea) [49, 50]. Unfortunately, the present mitogenomic phylogeny did not include representatives of Acuariidae or Acuarioidea, due to their mitogenomic data being currently unavailable. Moreover, it has also become apparent that 18S rDNA alone lacks the resolving power for clarifying deep phylogenetic relationships in many lineages of Nematoda [48]. Consequently, the familial allocation of *Crassicauda* remains an open question. This study just proposed that the Proleptidae stat. nov. seems to include the following five genera: *Heliconema*, *Paraleptus*, *Proleptus*, *Rasheedia*, and *Crassicauda*, but we do not make any immediate systematic change for *Crassicauda*, because a more rigorous mitogenomic phylogeny with broader representation of Spirurida, especially the superfamilies Acuarioidea and Habronematoidea, is required. Meanwhile, the Physalopteridae sensu stricto now comprises only two subfamilies, the Thubunaeinae and the Physalopterinae. Our phylogenetic results all support the validity of the subfamily Thubunaeinae, and the present phylogeny based on the 18S + *cox1* data indicates the transfer of the genus *Skrjabinoptera* from the Physalopterinae to the Thubunaeinae. In fact, the previous phylogeny based on the single 18S rRNA [51] also suggested a close affinity of *Skrjabinoptera* and *Physalopteroides*, which is accordant with this study.

According to traditional classifications [8–10, 12, 14], the superfamily Thelazioidea includes the Pneumospiruridae, Rhabdochonidae, and Thelaziidae. However, the phylogenetic relationships of these three families within the Thelazioidea remains uncertain [11, 47]. Due to the inaccessibility of suitable genetic data, the present phylogenies did not include representatives of the Rhabdochonidae. However, our phylogenetic results from the different datasets fail to support a close relationship between the Thelaziidae and the Pneumospiruridae, but suggest a close affinity between the Thelaziidae and the superfamily Filarioidea, as indicated by other recent molecular phylogenies [11, 16, 38, 47, 52, 53]. The phylogenetic status of the Pneumospiruridae within the Spirurida is not concordant with the present phylogenetic trees based on different datasets [Pneumospiruridae + (Physalopteridae sensu stricto + (Spiruroidea + (Thelaziidae + Filarioidea))) in the BI and ML trees of concatenated amino acid sequences of the 12PCGs, and nucleotide sequences of the 18S + 28S + 12PCGs and ML tree of 18S + *cox*1 data versus Pneumospiruridae + (Proleptidae stat. nov. + (Physalopteridae sensu stricto + (Spiruroidea + (Thelaziidae + Filarioidea)))) in only the BI tree based on the 18S + *cox*1 data]; however, all of the results show that the Pneumospiruridae has a distant relationship with the Thelaziidae and does not belong to the superfamily Thelazioidea. This is easy to understand when the different ecological niches of species of the Thelaziidae and Pneumospiruridae are considered. Members of the Thelaziidae generally occur in the orbital cavity of birds and domestic mammals [3]. In contrast, species of the Pneumospiruridae especially parasitize the respiratory system of mammals [8–10, 14]. The detailed systematic status of the Pneumospiruridae still needs to be clarified.

## Conclusions

The complete mitogenomes of *T. pudica* with 13,645 bp and *A. varani* with 13,730 bp are presented for the first time, which both include 36 genes and belong to the GA9 type gene arrangement. *Thubunaea pudica* is the first for the subfamily Thubunaeinae, and that of *A. varani* is also the first for the genus *Abbreviata*. Molecular phylogenetic results suggest to elevate the subfamily Proleptinae to full family level, Proleptidae *stat. nov*., and transfer the tetramerid genus *Crassicauda* to the Proleptidae *stat. nov*. Our results also indicate that the Physalopteridae *sensu stricto* currently comprises two subfamilies, the Thubunaeinae and the Physalopterinae, and that the genus *Skrjabinoptera* should be transferred from the Physalopterinae to the Thubunaeinae.

## Supplementary Information

Supplementary Material 1: Table S1. The primers and cycling conditions for amplifying different target regions using polymerase chain reactionin the present study.

Supplementary Material 2: Table S2. Detailed information for representatives of Spirurida included in the present phylogenetic analyses.

Supplementary Material 3: Table S3. The optimal amino acid or nucleotide substitution models selected for ML analyses.

Supplementary Material 4: Table S4. Organization of *Thubunaea pudica* and *Abbreviata varani* mitogenomes.

Supplementary Material 5: Table S5. Base composition and skewness in the mitogenomes of *Thubunaea pudica* and *Abbreviata varani*.

## Data Availability

Voucher specimens of *T. pudica* (HBNU–N–R20240815YL) and *A. varani* (HBNU–N–R20250530YL) were deposited in the College of Life Sciences, Hebei Normal University, China. The sequence data of *T. pudica* and *A. varani* obtained herein are deposited in the GenBank database http://www.ncbi.nlm.nih.gov. (*T. pudica*: PV818394 and PV818395 for 18S, PV916231 and PV916232 for 28S, PV819810 for *cox1*, PV925727 for the mitogenome; *A. varani*: PV864872 for 18S, PV818399 for 28S, PV888636 for *cox1*, PV925728 for the mitogenome).
