# Supplementary material for: Molecular phylogenies provide insights into the evolutionary relationships of the Spirurida (Nematoda), with special emphasis on the superfamily Physalopteroidea
Source: Parasit Vectors. 2025 Nov 10;18:453. doi: 10.1186/s13071-025-07097-z (PMC12604212; doi:10.1186/s13071-025-07097-z)
Supplement: Supplementary file 1 — Supplementary Material 1: Table S1. The primers and cycling conditions for amplifying different target regions using polymerase chain reactionin the present study. [file 13071_2025_7097_MOESM1_ESM.docx]

**Table S1.** The primers and cycling conditions for amplifying different target regions using polymerase chain reaction (PCR) in the present study. Asterisk indicates that the 28S data of *A. varani* failed to amplify by PCR, but was successfully assembled using GetOrganelle v1.7.7.0 based on genomic data.

| Gene | Species | Primer sequence (5′→3′) | Cycling condition | References |
| --- | --- | --- | --- | --- |
| 18S | *T. pudica* | 18S-F: CGCGAATRGCTCATTACAACAGC  18S-R: GGGCGGTATCTGATCGCC | 94 ℃ for 5 min | [1] |
|  |  |  | 94 ℃ for 30 s |  |
|  | *A. varani* |  | 72 ℃ for 70 s (30 cycles) |  |
|  |  |  | 72 ℃ for 7 min |  |
| 28S | *T. pudica* | 28S-F: AGCGGAGGAAAAGAAACTAA  28S-R: ATCCGTGTTTCAAGACGGG | 94 ℃ for 5 min | [2] |
|  |  |  | 94 ℃ for 30 s |  |
|  | *A. varani** |  | 72 ℃ for 70 s (30 cycles) |  |
|  |  |  | 72 ℃ for 7 min |  |
| *cox 1* | *T. pudica* | cox1-F: TTTTTTGGTCATCCTGAGGTTTAT  cox1-R: ACATAATGAAAATGACTAACAAC | 94 ℃ for 5 min | [3] |
|  |  |  | 94 ℃ for 30 s |  |
|  |  |  | 55 ℃ for 30 s |  |
|  |  |  | 72 ℃ for 45 s (30 cycles) |  |
|  |  |  | 72 ℃ for 7 min |  |
|  | *A. varani* | NTF: TGATTGGTGGTTTTGGTAA  NTR: ATAAGTACGAGTATCAATATC | 94 °C for 1 min | [4] |
|  |  |  | 94 °C for 1 min |  |
|  |  |  | 50 °C for 1 min |  |
|  |  |  | 72 °C for 1 min (40 cycles) |  |
|  |  |  | 72 °C for 10 min |  |

**References**

1. Floyd RM, Rogers AD, Lambshead PJD, Smith CR. Nematode‐specific PCR primers for the 18S small subunit rRNA gene. Mol Ecol Notes. 2005;5:611–2.
2. Nadler SA, Hudspeth DS. Ribosomal DNA and phylogeny of the Ascaridoidea (Nemata: Secernentea): implications for morphological evolution and classification. Mol Phylogenet Evol. 1998;10:221–36.

3. Lazarova SS, Malloch G, Oliveira CM, Hübschen J, Neilson R. Ribosomal and mitochondrial DNA analyses of *Xiphinema americanum*-group populations. J Nematol. 2006;38:404–10.

4. Casiraghi M, Anderson T, Bandi C, Bazzocchi C, Genchi C. A phylogenetic analysis of filarial nematodes: comparison with the phylogeny of *Wolbachia* endosymbionts. Parasitology. 2001;122:93–103.
