# Supplementary material for: Molecular phylogenies provide insights into the evolutionary relationships of the Spirurida (Nematoda), with special emphasis on the superfamily Physalopteroidea
Source: Parasit Vectors. 2025 Nov 10;18:453. doi: 10.1186/s13071-025-07097-z (PMC12604212; doi:10.1186/s13071-025-07097-z)
Supplement: Supplementary file 2 — Supplementary Material 2: Table S2. Detailed information for representatives of Spirurida included in the present phylogenetic analyses. [file 13071_2025_7097_MOESM2_ESM.docx]

**Table S2.** Detailed information for representatives of Spirurida included in the present phylogenetic analyses.

| Species | Mitogenome length (bp) | AT content (%) | Gene arrangement | GenBank  ID for Mitogenome | GenBank  ID for 18S | GenBank ID for 28S | GenBank ID for *cox*1 | References |
| --- | --- | --- | --- | --- | --- | --- | --- | --- |
| **Ingroup** |  |  |  |  |  |  |  |  |
| **Gnathostomatoidea** |  |  |  |  |  |  |  |  |
| Gnathostomatidae |  |  |  |  |  |  |  |  |
| *Gnathostoma nipponicum* | 14093 | 74.44 | GA26 | NC034239 | AB181157 | AB181157 | NC034239 | [1-2] |
| *G. spinigerum* | 14079 | 71.13 | GA26 | NC027726 | AB181155 | AB181155 | NC027726 | [1, 3] |
| *G. dolores* | 13809 | 70.55 | GA26 | NC032073 | LC848948 | AB181156 | NC032073 | [1, 2, 4] |
| *G. binucleatum* | 14067 | 71.45 | GA34 | NC080314 | Z96946 | AB181159 | NC080314 | [1, 5] |
| **Camallanoidea** |  |  |  |  |  |  |  |  |
| Camallanidae |  |  |  |  |  |  |  |  |
| *Camallanus lacustris* | 18935 | 75.90 | GA48 | NC070137 | DQ442663 |  | NC070137 | [6, 7] |
| *C. cotti* | 17901 | 70.74 | GA40 | NC036308 | EF180071 |  | NC036308 | [8, 9] |
| **Dracunculoidea** |  |  |  |  |  |  |  |  |
| Dracunculidae |  |  |  |  |  |  |  |  |
| *Dracunculus medinensis* | 14628 | 72.72 | GA14 | AP017682 | MK163617 |  | AP017682 | unpublished |
| Philometridae |  |  |  |  |  |  |  |  |
| *Clavinema parasiluri* | 14350 | 69.86 | GA55 | OM469014 | DQ076682 |  | OM469014 | [7] |
| *Philometra* sp. | 14963 | 71.26 | GA56 | OM469015 | KC342903 |  | OM469015 | [7] |
| *Philometroides sanguineus* | 14378 | 72.72 | GA50 | NC024931 | DQ442676 |  | NC024931 | [6, 10] |
| **Habronematoidea** |  |  |  |  |  |  |  |  |
| Tetrameridae |  |  |  |  |  |  |  |  |
| *Crassicauda magna* | 13605 | 74.97 | GA9 | OQ834322 | KX354835 |  | OQ834322 | [11, 12] |
| *Tetrameres grusi* | 13709 | 71.56 | GA9 | MW648425 | KX172117 |  | MW648425 | [13, 14] |
| **Thelazioidea** |  |  |  |  |  |  |  |  |
| Pneumospiruridae |  |  |  |  |  |  |  |  |
| *Metathelazia capsulata* | 13659 | 79.19 | GA9 | NC085271 | OP004065 | OP021869 | NC085271 | [15] |
| Thelaziidae |  |  |  |  |  |  |  |  |
| *Thelazia callipaeda* | 13666 | 74.77 | GA9 | AP017700 | MF795662 | KY476401 | AP017700 | [16-18] |
| **Physalopteroidea** |  |  |  |  |  |  |  |  |
| Physalopteridae |  |  |  |  |  |  |  |  |
| *Heliconema longissimum* | 13610 | 79.11 | GA11 | GQ332423 | JF803926 | NC016127 | NC016127 | [19, 20] |
| *Proleptus obtusus* |  |  |  |  | KY411575 |  | KY411574 | [21] |
| *Paraleptus chiloschyllii* |  |  |  |  | OK482082 |  | MZ958986 | [22] |
| *Abbreviata caucasica* |  |  |  |  | MN956824 |  | MT231294 | [23] |
| *Skrjabinoptera vietnanensis* |  |  |  |  | MW016952 |  | MW015829 | [24] |
| *Physaloptera rara* | 13735 | 72.48 | GA9 | MH931178 | MH938367 |  | MH931178 | unpublished |
| *P. clausa* | 13744 | 74.82 | GA9 | PP108232 | OR088573 |  | PP108232 | unpublished |
| *P. alata* |  |  |  |  | AY702703 |  | AY702703 | [25] |
| *P. amazonica* |  |  |  |  | MZ391893 |  | MZ391893 | [26] |
| *P. bispiculata* |  |  |  |  | MK312472 |  | MK312472 | unpublished |
| *P. mirandai* |  |  |  |  | MK309356 |  | MK309356 | unpublished |
| *P. retusa* |  |  |  |  | KT894817 |  | KT894817 | unpublished |
| *P. sibirica* |  |  |  |  | KT894806 |  | KT894806 | [27] |
| *Turgida turgida* |  |  |  |  | KT894805 |  | KT894805 | [28] |
| *Physalopteroides* sp. |  |  |  |  | KP338605 |  | KC130709 | [29, 30] |
| *Thubunaea pudica* | 13645 | 75.45 | GA9 | PV925727 | PV818394  PV818395 | PV916231  PV916232 | PV819810 | Present study |
| *Abbreviata varani* | 13730 | 74.71 | GA9 | PV925728 | PV864872 | PV818399 | PV888636 | Present study |
| **Spiruroidea** |  |  |  |  |  |  |  |  |
| Gongylonematidae |  |  |  |  |  |  |  |  |
| *Gongylonema pulchrum* | 13798 | 75.95 | GA9 | AP017685 | AB495401 | AB495401 | AP017685 | [31] |
| Spirocercidae |  |  |  |  |  |  |  |  |
| *Spirocerca lupi* | 13780 | 73.73 | GA9 | NC021135 | AY751497 | AY751500 | NC021135 | [32] |
| **Filarioidea** |  |  |  |  |  |  |  |  |
| Onchocercidae |  |  |  |  |  |  |  |  |
| *Setaria labiatopapillos* | 13950 | 78.95 | GA9 | NC044071 | KP760164 | KP760407 | NC044071 | [33, 34] |
| *S. digitata* | 13839 | 75.14 | GA9 | NC014282 | MN728217 | EF199753 | NC014282 | [35, 36] |
| *Dirofilaria repens* | 13678 | 76.05 | GA9 | KX265047 | KP760134 | KP760376 | KX265047 | [33, 37] |
| *D. immitis* | 13814 | 74.16 | GA9 | NC005305 | KP760133 | KP760375 | NC005305 | [33, 38] |
| *Onchocerca flexuosa* | 13672 | 74.17 | GA9 | NC016172 |  | OR741781 | NC016172 | [39] |
| *O. lupi* | 13766 | 73.38 | GA9 | MW266120 | KX853341 | KX853349 | MW266120 | [40, 41] |
| *O. volvulus* | 13747 | 73.30 | GA10 | NC001861 |  | GQ202198 | NC001861 | [42] |
| *O. ochengi* | 13744 | 73.22 | GA9 | AP017694 | KP760157 | KP760400 | AP017694 | [33] |
| *Dipetalonema gracile* | 13647 | 73.72 | GA9 | NC066230 | KP760130 | KP760372 | NC066230 | [33, 43] |
| *Litomosoides sigmodontis* | 13883 | 72.83 | GA62 | AP017689 | AF227233 | KP760384 | AP017689 | [33] |
| *Acanthocheilonema viteae* | 13724 | 73.54 | GA9 | NC016197 | KP760117 | KP760359 | NC016197 | [33, 39] |
| *Loa loa* | 13590 | 75.54 | GA9 | HQ186250 | XR002251421 | XM003142672 | HQ186250 | [39] |
| *Chandlerella quiscali* | 13757 | 77.67 | GA8 | NC014486 | JQ867042 |  | NC014486 | [39, 44] |
| *Mansonella perstans* | 13619 | 74.09 | GA9 | MT361687 | MN432520 | MN432520 | MT361687 | [45, 46] |
| *M. ozzardi* | 13681 | 74.29 | GA9 | NC082197 | KP760147 | MN432519 | NC082197 | [33, 45, 47] |
| *Wuchereria bancrofti* | 13636 | 74.62 | GA9 | NC016186 | AY843438 | EU370161 | NC016186 | [39, 48] |
| *Brugia pahangi* | 13675 | 74.70 | GA9 | JAAVKF010000141 | EU496884 | KP760363 | JAAVKF010000141 | [33, 49] |
| *B. timori* | 13658 | 75.90 | GA9 | AP017686 | KP760122 | KP760364 | AP017686 | [33] |
| *B. malayi* | 13657 | 75.46 | GA9 | MT149211 | KP760120 | XM043082196 | MT149211 | [33, 50] |
| **Outgroup** |  |  |  |  |  |  |  |  |
| **Oxyuroidea** |  |  |  |  |  |  |  |  |
| Oxyuridae |  |  |  |  |  |  |  |  |
| *Enterobius vermicularis* | 14010 | 71.17 | GA13 | EU281143 | HQ646164 | LC416069 | EU281143 | [51-53] |

**References**

1. Ando K, Tsunemori M, Akahane H, Tesana S, Hasegawa H, Chinzei Y. Comparative study on DNA sequences of ribosomal DNA and cytochrome *c* oxidase subunit 1 of mitochondrial DNA among five species of gnathostomes. J Helminthol. 2006;80:7–13.

2. Sun M-M, Liu G-H, Ando K, Woo H-C, Ma J, Sohn W-M, et al. Complete mitochondrial genomes of *Gnathostoma nipponicum* and *Gnathostoma sp.*, and their comparison with other *Gnathostoma* species. Infect Genet Evol. 2017;48:109–15.

3. Liu G-H, Shao R, Cai X-Q, Li W-W, Zhu X-Q. *Gnathostoma spinigerum* mitochondrial genome sequence: a novel gene arrangement and its phylogenetic position within the class Chromadorea. Sci Rep. 2015;5:12691.

4. Suzuki R, Tokiwa T, Kasahara T, Rengulbai K. *Gnathostoma doloresi* in domestic pigs in the Republic of Palau, 2020–2022. Parasitol Int. 2025;105:103001.

5. Diaz-Camacho SP, Logan R, Báez-Flores ME, Delgado-Vargas F, Prieto-Alvarado R, Vega-López IF, et al. Complete mitochondrial genome of *Gnathostoma binucleatum*. Microbiol Resour Announc. 2024;13:e00366-23.

6. Wijová M, Moravec F, Horák A, Lukeš J. Evolutionary relationships of Spirurina (Nematoda: Chromadorea: Rhabditida) with special emphasis on dracunculoid nematodes inferred from SSU rRNA gene sequences. Int J Parasitol. 2006;36:1067–75.

7. Zou H, Lei H-P, Chen R, Chen F-L, Li W-X, Li M, et al. Evolutionary rates of mitochondrial sequences and gene orders in Spirurina (Nematoda) are episodic but synchronised. Water Biol Secur. 2022;1:100033.

8. Nadler SA, Carreno RA, Mejía-Madrid H, Ullberg J, Pagan C, Houston R, et al. Molecular phylogeny of clade III nematodes reveals multiple origins of tissue parasitism. Parasitology. 2007;134:1421–42.

9. Zou H, Jakovlić I, Chen R, Zhang D, Zhang J, Li W-X, et al. The complete mitochondrial genome of parasitic nematode *Camallanus cotti*: extreme discontinuity in the rate of mitogenomic architecture evolution within the Chromadorea class. BMC Genomics. 2017;18:840.

10. Su Y-B, Kong S-C, Wang L-X, Chen L, Fang R. Complete mitochondrial genome of *Philometra carassii* (Nematoda: Philometridae). Mitochondrial DNA A. 2016;27:1397–8.

11. Wan X, Zheng J, Li W, Zeng X, Yang J, Hao Y, et al. Parasitic infections in the East Asian finless porpoise *Neophocaena asiaeorientalis* *sunameri* living off the Chinese Yellow/Bohai Sea coast. Dis Aquat Org. 2017;125:63–71.

12. Qiao Y, Ma X, Zhong S, Xing Y, Chen X, Chen B. The first complete mitochondrial genome of macroparasite *Crassicauda magna* (Nematoda: Spirurida) from *Neophocoena sunameri* in ningbo, China. Mitochondrial DNA B. 2021;6:3011–2.

13. Bertram MR, Hamer GL, Snowden KF, Hartup BK, Hamer SA. Coccidian parasites and conservation implications for the endangered whooping crane (*Grus americana*). PLoS One. 2015;10:e0127679.

14. Gao J-F, Mao R-F, Li Y, Sun Y-Y, Gao Z-Y, Zhang X-G, et al. Characterization of the mitochondrial genome of *Tetrameres grusi* and insights into the phylogeny of Spirurina. Int J Parasitol Parasites Wildl. 2022;17:35–42.

15. Aleix-Mata G, Arcenillas-Hernández I, de Ybáñez MRR, Martínez-Carrasco C, Montiel EE, Sánchez A. Complete mitochondrial genome of *Metathelazia capsulata* (Pneumospiruridae) and comparison with other Spiruromorpha species. Parasitol Res. 2024;123:3.

16. Zhang X, Shi YL, Wang ZQ, Duan JY, Jiang P, Liu RD, et al. Morphological and mitochondrial genomic characterization of eyeworms (*Thelazia callipaeda*) from clinical cases in central China. Front Microbiol. 2017;8:1335.

17. Zhang X, Shi YL, Han LL, Xiong C, Yi SQ, Jiang P, et al. Population structure analysis of the neglected parasite *Thelazia callipaeda* revealed high genetic diversity in Eastern Asia isolates. PLoS Negl Trop Dis. 2018;12:e0006165.

18. Čabanová V, Kocák P, Víchová B, Miterpáková M. First autochthonous cases of canine thelaziosis in Slovakia: a new affected area in Central Europe. Parasit Vectors. 2017;10:179.

19. Moravec F. Some aspects of the taxonomy and biology of adult spirurine nematodes parasitic in fishes: a review. Folia Parasitol. 2013;54:239–57.

20. Park J-K, Sultana T, Lee S-H, Kang S, Kim HK, Min G-S, et al. Monophyly of clade III nematodes is not supported by phylogenetic analysis of complete mitochondrial genome sequences. BMC Genomics. 2011;12:392.

21. Silva C, Veríssimo A, Cardoso P, Cable J, Xavier R. Infection of the lesser spotted dogfish with *Proleptus obtusus* Dujardin, 1845 (Nematoda: Spirurida) reflects ontogenetic feeding behaviour and seasonal differences in prey availability. Acta Parasitol. 2017;62:471–6.

22. Tang L-S, Gu X-H, Wang J-H, Ni X-F, Zhou K-F, Li L. Morphological and molecular characterization of *Paraleptus chiloscyllii* Yin & Zhang, 1983 (Nematoda: Physalopteridae) from the brownbanded bambooshark *Chiloscyllium punctatum* Müller & Henle (Elasmobranchii: Orectolobiformes). Parasitol Int. 2022;87:102511.

23. Laidoudi Y, Medkour H, Latrofa MS, Davoust B, Diatta G, Sokhna C, et al. Zoonotic *Abbreviata caucasica* in wild chimpanzees (*Pan troglodytes verus*) from Senegal. Pathogens. 2020;9:517.

24. An OV, Van Ha N, Greiman SE, Tram QA, Tuan PA, Binh TT. Description and molecular differentiation of a new *Skrjabinoptera* (Nematode: physalopteridae) from *Eutropis macularia* (Sauria: Scincidae) in North-Central Vietnam. J Parasitol. 2021;107:172–8.

25. Rentería-Solís Z, Ramilo DW, Schmäschke R, Gawlowska S, Correia J, Lopes F, et al. Morphological and Molecular Identification of *Physaloptera alata* (Nematoda: Spirurida) in a Booted Eagle (*Aquila pennata*) from Portugal. Animals. 2023;13:1669.

26. Maldonado Jr A, Simões R, São Luiz J, Costa-Neto S, Vilela R. A new species of *Physaloptera* (Nematoda: Spirurida) from *Proechimys gardneri* (Rodentia: Echimyidae) from the Amazon rainforest and molecular phylogenetic analyses of the genus. J Helminthol. 2020;94:e68.

27. Chen H-X, Zeng J-L, Gao Y-Y, Zhang D, Li Y, Li L. Morphology and genetic characterization of *Physaloptera sibirica* Petrow & Gorbunov, 1931 (Spirurida: Physalopteridae), from the hog-badger Arctonyx collaris Cuvier (*Carnivora: Mustelidae*), with molecular phylogeny of Physalopteridae. Parasit Vectors. 2023;16:227.

28. Smythe AB, Sanderson MJ, Nadler SA. Nematode small subunit phylogeny correlates with alignment parameters. Syst Biol. 2006;55:972–92.

29. Goswami U, Chaudhary A, Verma C, Singh H. Molecular and ultrastructure characterization of two nematodes (*Thelandros scleratus* and *Physalopteroides dactyluris*) based on ribosomal and mitochondrial DNA sequences. Helminthologia. 2016;53:165–71.

30. Prosser SW, Velarde‐Aguilar MG, León‐Règagnon V, Hebert PD. Advancing nematode barcoding: a primer cocktail for the cytochrome *c* oxidase subunit I gene from vertebrate parasitic nematodes. Mol Ecol Resour. 2013;13:1108–15.

31. Halajian A, Eslami A, Salehi N, Ashrafi-Helan J, Sato H. Incidence and genetic characterization of *Gongylonema pulchrum* in cattle slaughtered in Mazandaran Province, northern Iran. Iran J Parasitol. 2010;5:10–8.

32. Liu G-H, Wang Y, Song H-Q, Li M-W, Ai L, Yu X-L, et al. Characterization of the complete mitochondrial genome of *Spirocerca lupi*: sequence, gene organization and phylogenetic implications. Parasit Vectors. 2013;6:45.

33. Lefoulon E, Bain O, Bourret J, Junker K, Guerrero R, Cañizales I, et al. Shaking the tree: multi-locus sequence typing usurps current onchocercid (filarial nematode) phylogeny. PLoS Negl Trop Dis. 2015;9:e0004233.

34. Gao J-F, Hou M-R, Cui Y-C, Shi T-R. The complete mitochondrial genome of *Setaria labiatepapillosa* (Spirurida: Setariidae). Mitochondrial DNA B. 2019;4:1632–3.

35. Yatawara L, Wickramasinghe S, Nagataki M, Rajapakse R, Agatsuma T. Molecular characterization and phylogenetic analysis of *Setaria digitata* of Sri Lanka based on CO1 and 12S rDNA genes. Vet Parasitol. 2007;148:161–5.

36. Yatawara L, Wickramasinghe S, Rajapakse R, Agatsuma T. The complete mitochondrial genome of *Setaria digitata* (Nematoda: Filarioidea): Mitochondrial gene content, arrangement and composition compared with other nematodes. Mol Biochem Parasitol. 2010;173:32–8.

37. Yilmaz E, Fritzenwanker M, Pantchev N, Lendner M, Wongkamchai S, Otranto D, et al. Correction: The Mitochondrial Genomes of the Zoonotic Canine Filarial Parasites *Dirofilaria* *(Nochtiella)* *repens* and *Candidatus* Dirofilaria (Nochtiella) Honkongensis Provide Evidence for Presence of Cryptic Species. PLoS Negl Trop Dis. 2020;14:e0008347.

38. Hu M, Gasser R, El-Osta YA, Chilton N. Structure and organization of the mitochondrial genome of the canine heartworm, *Dirofilaria immitis*. Parasitology. 2003;127:37–51.

39. McNulty SN, Mullin AS, Vaughan JA, Tkach VV, Weil GJ, Fischer PU. Comparing the mitochondrial genomes of *Wolbachia*-dependent and independent filarial nematode species. BMC Genomics. 2012;13:145.

40. Lefoulon E, Giannelli A, Makepeace BL, Mutafchiev Y, Townson S, Uni S, et al. Whence river blindness? The domestication of mammals and host-parasite co-evolution in the nematode genus *Onchocerca*. Int J Parasitol. 2017;47:457–70.

41. Roe CC, Urbanz J, Andrews L, Verocai GG, Engelthaler DM, Hepp CM, et al. Complete mitochondrial genome of *Onchocerca lupi* (Nematoda, Onchocercidae). Mitochondrial DNA B. 2021;6:2572–4.

42. Keddie EM, Higazi T, Unnasch TR. The mitochondrial genome of *Onchocerca volvulus*: sequence, structure and phylogenetic analysis. Mol Biochem Parasitol. 1998;95:111–27.

43. Costa CHA, Crainey JL, Vicente ACP, Conga DF, Gordo M, Luz SLB, et al. Ribosomal, mitochondrial and bacterial (*Wolbachia*) reference sequences for *Dipetalonema gracile* obtained from a wild pied tamarin (*Saguinus bicolor*) host in Manaus, Brazil. Acta Amaz. 2023;53:130–40.

44. Hamer GL, Anderson TK, Berry GE, Makohon-Moore AP, Crafton JC, Brawn JD, et al. Prevalence of filarioid nematodes and trypanosomes in American robins and house sparrows, Chicago USA. Int J Parasitol Parasites Wildl. 2013;2:42–9.

45. Crainey JL, Costa CHA, de Oliveira Leles LF, Ribeiro da Silva TR, de Aquino Narzetti LH, Serra dos Santos YV, et al. Deep sequencing reveals occult mansonellosis coinfections in residents from the Brazilian Amazon village of São Gabriel da Cachoeira. Clin Infect Dis. 2020;71:1990–3.

46. Chung M, Aluvathingal J, Bromley RE, Nadendla S, Fombad FF, Kien CA, et al. Complete mitochondrial genome sequence of *Mansonella perstans*. Microbiol Resour Announc. 2020;9(30):e00490-20. doi:10.1128/mra.00490-20.

47. Dahmer KJ, Palma-Cuero M, Ciuoderis K, Patiño C, Roitman S, Li Z, et al. Molecular surveillance detects high prevalence of the neglected parasite *Mansonella ozzardi* in the Colombian Amazon. J Infect Dis. 2023;228:1441–51.

48. Bhandari Y, Dabir P, Nandhakumar K, Dayananda KM, Shouche YS, Reddy MVR. Analysis of polymorphism of 18S rRNA gene in *Wuchereria bancrofti* microfilariae. Microbiol Immunol. 2005;49:909–14.

49. Fong M, Thanabalan A, Muslim A, Lau Y-L, Sivanandam S, Mahmud R. Inferring the phylogenetic position of *Brugia pahangi* using 18S ribosomal RNA (18S rRNA) gene sequence. Trop Biomed. 2008;25(1):87–92.

50. Qing X, Kulkeaw K, Wongkamchai S, Tsui SK-W. Mitochondrial genome of *Brugia malayi* microfilariae isolated from a clinical sample. Front Ecol Evol. 2021;9:637805.

51. Kang S, Sultana T, Eom KS, Park YC, Soonthornpong N, Nadler SA, et al. The mitochondrial genome sequence of *Enterobius vermicularis* (Nematoda: Oxyurida)—an idiosyncratic gene order and phylogenetic information for chromadorean nematodes. Gene. 2009;429:87–97.

52. Zelck UE, Bialek R, Weiß M. Molecular phylogenetic analysis of *Enterobius vermicularis* and development of an 18S ribosomal DNA-targeted diagnostic PCR. J Clin Microbiol. 2011;49:1602–4.

53. Frias L, Hasegawa H, Stark DJ, Lynn MS, Nathan SK, Chua TH, et al. A pinworm's tale: The evolutionary history of *Lemuricola (Protenterobius) nycticebi*. Int J Parasitol Parasites Wildl. 2019;8:25–32.
