# Supplementary material for: Molecular phylogenies provide insights into the evolutionary relationships of the Spirurida (Nematoda), with special emphasis on the superfamily Physalopteroidea
Source: Parasit Vectors. 2025 Nov 10;18:453. doi: 10.1186/s13071-025-07097-z (PMC12604212; doi:10.1186/s13071-025-07097-z)
Supplement: Supplementary file 3 — Supplementary Material 3: Table S3. The optimal amino acid or nucleotide substitution models selected for ML analyses. [file 13071_2025_7097_MOESM3_ESM.docx]

**Table S3.** The optimal amino acid or nucleotide substitution models selected for phylogenetic analyses.

| Genetic data | Optimal substitution models |
| --- | --- |
| amino acid (AA) sequences of 12 PCGs | JTT + F + I + G4 |
| nucleotide sequences of 18S + 28S + 12 PCGs | GTR + F + I + G4 |
| nucleotide sequences of 18S + *cox*1 | Blosum62 + F + I for 18S |
|  | mtZOA + F + R4 for *cox1* |
