# Supplementary material for: Molecular phylogenies provide insights into the evolutionary relationships of the Spirurida (Nematoda), with special emphasis on the superfamily Physalopteroidea
Source: Parasit Vectors. 2025 Nov 10;18:453. doi: 10.1186/s13071-025-07097-z (PMC12604212; doi:10.1186/s13071-025-07097-z)
Supplement: Supplementary file 4 — Supplementary Material 4: Table S4. Organization of Thubunaea pudica and Abbreviata varani mitogenomes. [file 13071_2025_7097_MOESM4_ESM.docx]

**Table S4.** Organization of *Thubunaea pudica* and *Abbreviata varani* mitogenomes. “Ini/Ter cod” and “Int seq” representing initial/terminal codons and the length of intergenic sequences, respectively.

| Gene/Region | Position 5’ to 3’ | Length (bp) | Ini/Ter cod | Anticodon | Strand | Int seq |
| --- | --- | --- | --- | --- | --- | --- |
| ***Thubunaea pudica*** | |  |  |  |  |  |
| *cox*1 | 1-1647 | 1647 | ATG/ TAA |  | + | 6 |
| *trn*W | 1654-1710 | 57 |  | uca | + | 3 |
| *nad*6 | 1714-2178 | 465 | ATT/ TAA |  | + | 0 |
| *trn*R | 2179-2232 | 54 |  | acg | + | 0 |
| *trn*Q | 2233-2286 | 54 |  | uug | + | 0 |
| *cytb* | 2287-3375 | 1089 | ATA/ TAA |  | + | 2 |
| *trn*L1 | 3378-3434 | 57 |  | uag | + | -1 |
| *cox*3 | 3434-4207 | 774 | ATT/ TAA |  | + | 383 |
| *trn*A | 4591-4645 | 55 |  | ugc | + | 6 |
| *trn*L2 | 4652-4706 | 55 |  | uaa | + | 0 |
| *trn*N | 4707-4762 | 56 |  | guu | + | 0 |
| *trn*M | 4763-4818 | 56 |  | cau | + | 1 |
| *trn*K | 4820-4876 | 57 |  | uuu | + | 13 |
| *nad*4L | 4890-5108 | 219 | ATT/ TAG |  | + | 0 |
| *rrn*S | 5109-5791 | 683 |  |  | + | 0 |
| *trn*Y | 5792-5843 | 52 |  | gua | + | 0 |
| *nad*1 | 5844-6730 | 887 | TTG/ TA |  | + | 0 |
| *trn*F | 6731-6782 | 52 |  | gaa | + | -1 |
| *atp*6 | 6782-7366 | 585 | ATG/ TAA |  | + | 0 |
| *trn*I | 7367-7422 | 56 |  | gau | + | 2 |
| *trn*G | 7425-7481 | 57 |  | ucc | + | 6 |
| *cox*2 | 7488-8171 | 684 | ATA/ TAA |  | + | 0 |
| *trn*H | 8172-8228 | 57 |  | gug | + | 0 |
| *rrn*L | 8229-9170 | 942 |  |  | + | 0 |
| *nad*3 | 9171-9509 | 339 | ATT/ TAA |  | + | 0 |
| *trn*C | 9510-9562 | 53 |  | gca | + | -1 |
| *trn*S2 | 9562-9613 | 52 |  | uga | + | 0 |
| *trn*P | 9614-9668 | 55 |  | ugg | + | 43 |
| *trn*D | 9712-9768 | 57 |  | guc | + | 2 |
| *trn*V | 9771-9826 | 56 |  | uac | + | -1 |
| *nad*5 | 9826-11401 | 1576 | TTG/ T |  | + | 0 |
| *trn*E | 11402-11453 | 52 |  | uuc | + | -1 |
| *trn*S1 | 11453-11503 | 51 |  | acu | + | -1 |
| *nad*2 | 11503-12357 | 855 | ATT/ TAG |  | + | 0 |
| *trn*T | 12358-12414 | 57 |  | ugu | + | 0 |
| *nad*4 | 12415-13644 | 1230 | ATA/ TAA |  | + | 1 |
| ***Abbreviata varani*** | |  |  |  |  |  |
| *cox1* | 1-1557 | 1557 | ATT/ TAA |  | + | 1 |
| *trn*W | 1559-1613 | 55 |  | uca | + | -2 |
| *nad*6 | 1612-2064 | 453 | TTG/ TAG |  | + | -2 |
| *trn*R | 2063-2118 | 56 |  | acg | + | 0 |
| *trn*Q | 2119-2173 | 55 |  | uug | + | 12 |
| *cytb* | 2186-3258 | 1073 | ATA/ TA |  | + | -1 |
| *trn*L1 | 3258-3314 | 57 |  | uag | + | -3 |
| *cox*3 | 3312-4085 | 774 | ATG/ TAA |  | + | 544 |
| *trn*A | 4630-4685 | 56 |  | ugc | + | 5 |
| *trn*L2 | 4691-4744 | 54 |  | uaa | + | 0 |
| *trn*N | 4745-4798 | 54 |  | guu | + | 4 |
| *trn*M | 4803-4862 | 60 |  | cau | + | 1 |
| *trn*K | 4864-4919 | 56 |  | uuu | + | 3 |
| *nad*4L | 4923-5198 | 276 | ATT/ TAG |  | + | 7 |
| *rrn*S | 5206-5841 | 636 |  |  | + | -2 |
| *trn*Y | 5840-5895 | 56 |  | gua | + | 9 |
| *nad*1 | 5905-6754 | 850 | ATG/ T |  | + | 6 |
| *trn*F | 6761-6816 | 56 |  | gaa | + | 0 |
| *aot*6 | 6817-7398 | 582 | TTG/ TAA |  | + | -2 |
| *trn*I | 7397-7454 | 58 |  | gau | + | 5 |
| *trn*G | 7460-7514 | 55 |  | ucc | + | -1 |
| *cox*2 | 7514-8206 | 693 | ATG/ TAG |  | + | -2 |
| *trn*H | 8205-8258 | 54 |  | gug | + | 0 |
| *rrn*L | 8259-9213 | 955 |  |  | + | 0 |
| *nad*3 | 9214-9540 | 327 | ATT/ TAG |  | + | -2 |
| *trn*C | 9539-9593 | 55 |  | gca | + | 0 |
| *trn*S2 | 11473-11526 | 54 |  | uga | + | 1 |
| *trn*P | 9645-9698 | 54 |  | ugg | + | 10 |
| *trn*D | 9709-9763 | 55 |  | guc | + | 2 |
| *trn*V | 9766-9820 | 55 |  | uac | + | 3 |
| *nad*5 | 9824-11414 | 1591 | ATT/ T |  | + | 3 |
| *trn*E | 11418-11473 | 56 |  | uuc | + | -1 |
| *trn*S1 | 11473-11526 | 54 |  | acu | + | 12 |
| *nad*2 | 11539-12351 | 813 | ATA/ TAG |  | + | -1 |
| *trn*T | 12351-12405 | 55 |  | ugu | + | -1 |
| *nad*4 | 12405-13634 | 1230 | ATG/ TAG |  | + | 1 |
