# Supplementary material for: Molecular phylogenies provide insights into the evolutionary relationships of the Spirurida (Nematoda), with special emphasis on the superfamily Physalopteroidea
Source: Parasit Vectors. 2025 Nov 10;18:453. doi: 10.1186/s13071-025-07097-z (PMC12604212; doi:10.1186/s13071-025-07097-z)
Supplement: Supplementary file 5 — Supplementary Material 5: Table S5. Base composition and skewness in the mitogenomes of Thubunaea pudica and Abbreviata varani. [file 13071_2025_7097_MOESM5_ESM.docx]

**Table S5.** Base composition and skewness of the mitogenomes of *Thubunaea pudica* and *Abbreviata varani*

| Location | A% | T% | C% | G% | AT% | AT-skew | GC-skew | size (bp) |
| --- | --- | --- | --- | --- | --- | --- | --- | --- |
| ***Thubunaea pudica*** |  |  |  |  |  |  |  |  |
| Whole mitochondrial genome | 24.94 | 50.51 | 6.57 | 17.98 | 75.45 | -0.34 | 0.46 | 13645 |
| Protein coding genes (PCGs) | 22.08 | 51.79 | 7.02 | 19.11 | 73.86 | -0.40 | 0.46 | 10350 |
| 1st codon | 26.14 | 44.16 | 7.45 | 22.25 | 70.30 | -0.26 | 0.50 | 3451 |
| 2nd codon | 18.61 | 51.88 | 11.19 | 18.32 | 70.49 | -0.47 | 0.24 | 3450 |
| 3rd codon | 21.48 | 59.32 | 2.44 | 16.76 | 80.81 | -0.47 | 0.75 | 3449 |
| tRNAs | 35.02 | 45.86 | 4.39 | 14.74 | 80.88 | -0.13 | 0.54 | 1208 |
| rRNAs | 32.12 | 46.40 | 5.72 | 15.75 | 78.52 | -0.18 | 0.47 | 1625 |
| *rrn*S | 33.09 | 43.92 | 5.56 | 17.42 | 77.01 | -0.14 | 0.52 | 683 |
| *rrn*L | 31.42 | 48.20 | 5.84 | 14.54 | 79.62 | -0.21 | 0.43 | 942 |
| Non-coding region (NCR) | 40.21 | 48.30 | 4.44 | 7.05 | 88.51 | -0.09 | 0.23 | 383 |
| ***Abbreviata varani*** |  |  |  |  |  |  |  |  |
| Whole mitochondrial genome | 26.38 | 48.33 | 6.66 | 18.62 | 74.71 | -0.29 | 0.47 | 13730 |
| Protein coding genes (PCGs) | 23.35 | 49.25 | 7.23 | 20.17 | 72.60 | -0.36 | 0.47 | 10219 |
| 1st codon | 27.05 | 42.49 | 7.57 | 22.89 | 69.54 | -0.22 | 0.50 | 3408 |
| 2nd codon | 18.85 | 51.15 | 10.86 | 19.14 | 69.99 | -0.46 | 0.28 | 3406 |
| 3rd codon | 24.14 | 54.13 | 3.26 | 18.47 | 78.27 | -0.38 | 0.70 | 3405 |
| tRNAs | 36.54 | 44.17 | 3.77 | 15.52 | 80.70 | -0.09 | 0.61 | 1166 |
| rRNAs | 32.94 | 45.63 | 5.47 | 15.96 | 78.57 | -0.16 | 0.49 | 1591 |
| *rrnS* | 33.81 | 44.81 | 5.66 | 15.72 | 78.62 | -0.14 | 0.47 | 636 |
| *rrnL* | 32.36 | 46.18 | 5.34 | 16.13 | 78.53 | -0.18 | 0.50 | 955 |
| Long non-coding region (LNCR) | 40.81 | 45.96 | 6.25 | 6.99 | 86.77 | -0.06 | 0.06 | 544 |
| Short non-coding region (SNCR) | 39.58 | 44.79 | 6.25 | 9.38 | 84.37 | -0.06 | 0.20 | 96 |
